# Predicting Deliquescence Relative Humidities of Crystals and Crystal Mixtures

**DOI:** 10.3390/molecules26113176

**Published:** 2021-05-26

**Authors:** Heiner Veith, Christian Luebbert, Gabriele Sadowski

**Affiliations:** Laboratory of Thermodynamics, Department of Chemical and Biochemical Engineering, TU Dortmund University, Emil-Figge-Str. 70, D-44227 Dortmund, Germany; heiner.veith@tu-dortmund.de (H.V.); christian.luebbert@tu-dortmund.de (C.L.)

**Keywords:** crystal, crystal mixture, vapor sorption, deliquescence, PC-SAFT, thermodynamics

## Abstract

The presence of water in the form of relative humidity (RH) may lead to deliquescence of crystalline components above a certain RH, the deliquescence RH (DRH). Knowing the DRH values is essential, e.g., for the agrochemical industry, food industry, and pharmaceutical industry to identify stability windows for their crystalline products. This work applies the Perturbed-Chain Statistical Associating Fluid Theory (PC-SAFT) to purely predict the DRH of single components (organic acids, sugars, artificial sweeteners, and amides) and multicomponent crystal mixtures thereof only based on aqueous solubility data of the pure components. The predicted DRH values very well agree with the experimental ones. In addition, the temperature influence on the DRH value could be successfully predicted with PC-SAFT. The DRH prediction also differentiates between formation of hydrates and anhydrates. PC-SAFT-predicted phase diagrams of hydrate-forming components illustrate the influence of additional components on the hydrate formation as a function of RH. The DRH prediction via PC-SAFT allows for the determining of the stability of crystals and crystal mixtures without the need for time-consuming experiments.

## 1. Introduction

Water is omnipresent in the atmosphere and might be potentially absorbed by any kind of product. A certain relative humidity (RH) can thus have a huge influence on pharmaceutical, agrochemical, and food products. It can lead to changes in physical stability, chemical reactivity (loss of shelf life), and microbial stability [1]. The mechanisms of water-product interactions range widely from adsorption onto the surface of solids, via condensation of water in capillaries of the solid or condensation of water to form a saturated solution with the solid (deliquescence), to molecularly incorporated water in the form of absorbed water (e.g. by polymers), and formation of crystal hydrates [2]. Deliquescence leads to the most significant changes in physical properties of a crystalline solid [2]. Deliquescence is a first-order phase transformation, where a crystalline solid is transformed into an aqueous saturated solution. This occurs abruptly as soon as a crystal is stored above its deliquescence relative humidity (DRH) [2]. 

In principle, each crystalline compound has a DRH, above which it dissolves. Deliquescence values are reported for inorganic salts [3], organic salts [4,5], organic acids/ bases [6,7], vitamins [8], and sugars [7] since these compounds might reveal low DRH values, which are highly relevant for storage stability. Crystalline solids of high aqueous solubility usually show lower DRHs [2]. Deliquescence may also be induced by organic solvent vapors. In this case, deliquescence is denoted as ‘vapor digestion’ [9] in the literature. Nevertheless, water deliquescence is more common due to the omnipresent RH in the atmosphere.

The measurement of DRH is performed via gravimetric vapor-sorption measurements, equilibrium water-activity measurements of saturated solutions, or determining dynamic dew-point sorption profiles [10]. Dynamic measurements, e.g., of gravimetric vapor-sorption and dew-point-sorption profiles might be error prone due to kinetic factors (i.e., thermodynamic equilibrium not reached). Water-activity measurements of saturated solutions are more reliable since these are true equilibrium measurements. However, they also might be error prone due to polymorphic changes or chemical reactions upon substance dissolution in water [10]. Therefore, extensive investigations of the phase behavior are worthwhile. Li et al. investigated the deliquescence kinetics of crystals and crystal mixtures and determined the DRH through the extrapolation vapor-sorption rate to 0, where the deliquescence is about to occur [11]. Recently, the DRH of deep eutectic systems was experimentally investigated, showing that deliquescence via RH leads to liquification, although the deep eutectic solvent would be solid without the presence of RH [12]. Predictions of the DRH values for single components have been performed via Raoult’s law. This simplest approach neglects any solute–water interactions in the liquid phase, and results might significantly deviate from experimental data [13]. UNIFAC correlations [6,7] work well for some organic crystals [7], but DRH values e.g. of dicarboxylic acids differ by about 11–104% from the experimental data [6]. 

In multicomponent crystal mixtures, where crystals of different substances are in contact with each other, the DRH of the crystal mixture (DRH_mix_; also called RH_0mix [14]_) lies below the DRH of any of the individual crystal [15]. The prediction of this decrease in DRH was frequently investigated in References [16,17]. For example, Ross et al. developed a simple equation to obtain the DRH_mix_, which however neglects the interactions between the different solutes in the liquid phase [17]. The predictions with the Ross equation are always based on single-component DRH values, which have to be experimentally determined beforehand. The deviation of the predicted DRH_mix_ from the experimental one was found to be up to 50% for multicomponent crystal mixtures [5]. Several attempts to improve the Ross equation were found to be only satisfactory for some crystal mixtures but did not improve the DRH predictions in general [18]. 

The scope of this work is to predict the DRH values based on a minimum of experimental data with a reasonably good agreement to the measured DRH values, rather than performing additional experiments and model fittings to exactly meet the measured DRH values. DRH values of single components and of multicomponent crystal mixtures were predicted using PC-SAFT only based on the single-component solubility data in water. Furthermore, the influence of temperature on the DRH was predicted and compared with measured DRH values from literature.

## 2. Theory

Three phases (vapor in form of relative humidity, crystals, and saturated liquid) are present as soon as the DRH of a crystal is exceeded. The calculation of the thermodynamic equilibrium between all three phases allows one to determine the DRH. The crystalline component(s) is(are) only present in the solid phase and in the liquid phase, whereas water is present in the liquid phase and the vapor phase. Thus, the solid–liquid equilibrium has to be solved for the crystalline component (or multiple crystalline components in mixtures; hereafter referred to as crystal mixtures). Simultaneously, the vapor–liquid equilibrium for water has to be solved.

### 2.1. Solid–Liquid Equilibrium

The solid–liquid equilibrium is used to calculate the crystalline-component solubility, where the chemical potential of the component *i* is the same in the solid and the liquid phase. The mole fraction solubilities $x_{i}^{SL}$ of the component *i* are calculated by Equation (1) [19].

(1) $$x_{i}^{SL}=\frac{1}{\gamma_{i}}\exp\left[-\frac{\Delta h_{i}^{SL}}{R\cdot T}\cdot \left(1-\frac{T}{T_{i}^{SL}}\right)-\frac{\Delta c_{p,i}^{SL}}{R}\cdot \left(ln\left(\frac{T_{i}^{SL}}{T}\right)-\frac{T_{i}^{SL}}{T}+1\right)\right]$$

In this equation, $T_{i}^{SL}$, $\Delta h_{i}^{SL}$, and $\Delta c_{p,i}^{SL}$ are the melting temperature, melting enthalpy, and heat capacity change upon melting of component *i*. R is the ideal gas constant, and T is the temperature at which the solubility is to be determined. The activity coefficient $\gamma_{i}$ explicitly considers the interactions between all components present in the liquid phase. 

The solubility product $K_{s,hydrate}$ was applied to determine the solubility of hydrate-forming components *i*. Equation (2) was used to determine the solubility $x_{i}S^{L}$ of the component iteratively, since the activity coefficients also depend on concentration.

(2) $$K_{s,hydrate}={\left(x_{i}^{SL}\cdot \gamma_{i}\right)}^{\nu_{i}}\cdot {\left(x_{water}\cdot \gamma_{water}\right)}^{\nu_{water}}$$

The solubility product of highly water-soluble hydrates considered in this work was determined by Equation (3). For that purpose, the previously published equation [20] was extended in this work by the water activity at the transition between hydrate and anhydrate $a_{water}^{trans}$. In the case of hydrates (or solvates, which are not considered in this work) with a very low solubility, this value is almost unity, and Equation (3) again reduces to the previously published version. The derivation of Equation (3) can be found in the supporting information (Equations (S1)–(S4)).

(3) $$K_{s,hydrate}={\left(exp\left[-\frac{\Delta h_{i}^{SL}}{R\cdot T}\left(1-\frac{T}{T_{i}^{SL}}\right)-\frac{\Delta c_{P,i}^{SL}}{R}\left(ln\left(\frac{T_{i}^{SL}}{T}\right)-\frac{T_{i}^{SL}}{T}+1\right)\right]\right)}^{\nu_{i}}\;\cdot \;{\left(a_{water}^{trans}\cdot exp\left[\frac{-\Delta h^{trans}}{\nu_{water}\cdot R\cdot T}\left(1-\frac{T}{T^{trans}}\right)\right]\right)}^{\nu_{water}}$$

The melting properties of the anhydrate (see Equation (1)), the enthalpy of transition (upon dehydration) $\Delta h^{trans}$, the transition temperature $T^{trans}$, the water activity at transition $a_{water}^{trans}$ (only for highly water-soluble hydrates), and the stoichiometry of the hydrate ($\nu_{i}$, $\nu_{water}$) are used to determine the temperature-dependent solubility product. The solubility of metastable crystals can be considered by using melting data of the metastable polymorph (Equation (1)) or the solubility product of the (in this case) metastable hydrate (Equation (2)). 

The activity coefficients can be calculated using a thermodynamic model (in this work PC-SAFT) or set to unity (assumption of Raoult’s law). The calculation of solubilities with Raoult’s law would lead to significant discrepancies between the calculated solubility and the measured one. In particular, neglecting the activity coefficients using Raoult’s law would imply that the solubility of a crystalline component does not depend on the solvent. To allow better comparison of the models for subsequent calculations, the mole fraction solubilities of the crystallizing components $x_{i}^{SL}$ in water were always obtained from PC-SAFT calculations, which were previously fitted to experimental solubility data.

### 2.2. Vapor-Liquid Equilibrium

The equilibrium of water vapor in the atmosphere with the liquid phase was calculated by Equation (4). This equation assumes that the vapor phase behaves like an ideal gas.

(4) $$\frac{\mathrm{RH}}{100\%}=\frac{p_{water}}{p_{water}^{LV}}=x_{water}\cdot \gamma_{water}=a_{water}$$

The RH in the system is determined by the partial pressure of water in the vapor phase ($p_{water}$) divided by the saturation vapor pressure of water at the given temperature ($p_{water}^{LV}$). The mole fraction of water in the liquid phase in equilibrium with the vapor phase is determined by the activity coefficient of water ($\gamma_{water}$), which depends on the composition of every component in the liquid phase. The product of $x_{water}$ and $\gamma_{water}$ is the water activity ($a_{water}$), which in thermodynamic equilibrium equals the RH. 

### 2.3. Calculation of Deliquescence Relative Humidity

DRH values of single crystals (DRH_i_) and crystal mixtures (DRH_mix_) can be predicted by solving the vapor–liquid–solid equilibrium while considering the single crystal or several crystals simultaneously as solid phase. The solid–liquid equilibrium in Equation (1) yields the mole fractions of the crystallizing component(s) in a saturated solution. For a multicomponent crystal mixture, the solid–liquid equilibrium of each crystal must be solved individually while considering the presence of the other dissolved components in the liquid phase. Thus, the overall concentrations of components in the liquid phase correspond to a liquid phase, which is in equilibrium with each crystal of the crystal mixture resulting in the eutectic composition (liquid phase is saturated with respect to every component of the crystal mixture). 

The water concentration in this saturated solution is calculated from Equation (5) using the equilibrium solubility mole fractions of all solutes $x_{i}^{SL}$ simultaneously determined using Equation (1) (or Equation (2) for hydrates).

(5) $$x_{water}=1-\underset{i\neq water}{\sum }x_{i}^{SL}$$

The water activity ($a_{water}$), which is determined additionally using the water activity coefficient (see Equation (4)), directly yields the DRH of the crystallizing component.

Calculation of the DRH via the Raoult’s law yields:

(6) $$\frac{{\mathrm{DRH}}_{\mathrm{Raoult}}}{100\%}=x_{water}$$

### 2.4. PC-SAFT

The Perturbed-Chain Statistical Associating Fluid Theory (PC-SAFT) [21,22] is a model for the residual Helmholtz energy. The latter is assumed to be composed out of different contributions, namely, the hard-chain repulsion $A^{hard-chain}$, dispersive attractions such as Van der Waals forces $A^{dispersion}$ and hydrogen-bond formation $A^{association}$ (Equation (7)).

(7) $$A^{residual}=A^{hard-chain}+A^{dispersion}+A^{association}$$

Five pure-component parameters are required to describe pure components, namely segment number ($m_{seg}$), segment diameter ($\sigma_{i}$), dispersion energy ($u_{i}/k_{B}$), association energy ($\epsilon^{A_{i}B_{i}}/k_{B}$), and association volume ($\kappa^{A_{i}B_{i}}$). Mixing rules are applied to describe mixtures. The segment diameter $\sigma_{ij}$ (Equation (8)) and dispersion energy $u_{ij}$ (Equation (9)) of a mixture were determined by Lorentz–Berthelot combining rules [23,24].

(8) $$\sigma_{ij}=\frac{1}{2}\left(\sigma_{i}+\sigma_{j}\right)$$

(9) $$u_{ij}=\sqrt{u_{i}u_{j}}\cdot \left(1-k_{ij}\right)$$

Combining rules of Wolbach and Sandler were used to determine the association energy $\varepsilon^{AiBj}$ (Equation (10)) and association volume $\kappa^{AiBj}$ (Equation (11)) in mixtures [25].

(10) $$\varepsilon^{AiBj}=\frac{1}{2}\left(\varepsilon^{AiBi}+\varepsilon^{AjBj}\right)$$

(11) $$\kappa^{AiBj}=\sqrt{\kappa^{AiBi}\kappa^{AjBj}}{\left(\frac{\sqrt{\sigma_{i}\sigma_{j}}}{\left(1/2\right)\left(\sigma_{i}+\sigma_{j}\right)}\right)}^{3}$$

The binary interaction parameter in Equation (9) was fitted to experimental data and is either a constant or linearly temperature-dependent according to Equation (12).

(12) $$k_{ij}=k_{ij,T}\cdot T+k_{ij,b}$$

Table 1 and Table 2 show the pure-component parameters and binary interaction parameters used in this work. The melting properties of the components used in this work are shown in Table 3.

The $k_{ij}$ between citric acid (CA) and water was fitted to solubility data of the hydrate and anhydrate at temperatures between 293.15 and 323.15 K. The CA hydrate solubility was calculated using the solubility product (Equation (2)) with a transition enthalpy of 10.85 kJ mol^−1^ [32], a transition temperature of 309.45 K [33], and a modeled hydrate transition water activity of 0.725. All other $k_{ij}$ values of this work were obtained from the literature. Table 2 also lists the type of experimental data which the $k_{ij}$s were fitted to. Osmotic coefficients of aqueous solutions are directly linked to the water activity in the solution [29]. The $k_{ij}$-values for the systems ascorbic acid/water, fructose/water, lactose/water, and sucrose/water were fitted to osmotic coefficients of (unsaturated) aqueous solutions [27,29]. Binary interaction parameters between solute components in mixtures were set to zero to keep this approach predictive. Certainly, a binary interaction parameter between the crystalline components in crystal mixtures would even better describe the solubility of crystal mixtures in water, and the DRH_mix_ prediction would also improve.

## 3. Results and Discussion

### 3.1. DRH Prediction of Single Components

**Figure 1:**
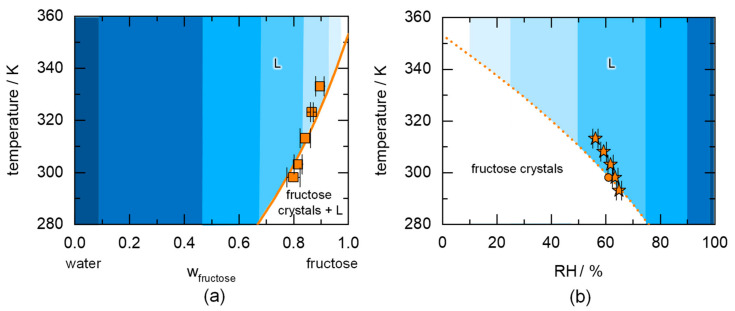
Temperature-dependent binary-phase diagrams indicating (**a**) the solubility of crystalline fructose in water and (**b**) the DRH of fructose. The solid line is the PC-SAFT-predicted equilibrium solubility of fructose in water (**a**) and the dotted line is the PC-SAFT-predicted DRH of fructose as a function of temperature (**b**). Darker-shaded regions indicate higher RH values, whereas brighter-shaded regions indicate lower RH regions. The squares in (**a**) are experimental data for fructose solubility [43]. The stars in (**b**) are DRH values measured via vapor-sorption measurement [44] and the circle is the measured equilibrium water activity of a saturated solution [14].

The deliquescence phenomenon is directly related to the solubility of the component. Figure 1a shows the temperature-dependent aqueous solubility of the example component fructose. The example system fructose/water is used to explain the use of the phase diagrams and serves as a proof of principle for the PC-SAFT predictions. In this diagram, pure fructose and pure water are present on the right and left axis of the diagram, respectively. The high solubility of fructose increases with temperature reaching the melting point of fructose at 353.2 K. Although the binary interaction parameter was fitted to the osmotic coefficient (related to the water activity), the PC-SAFT calculated solubility of fructose very well agrees with the experimental solubility data. Due to hydrate formation of fructose [42], solubility data below 298.15 K is not considered in this work. 

The two diagrams in Figure 1 were calculated using (a) the solid–liquid equilibrium (Equation (1)) for fructose and (b) the solid–liquid–vapor equilibrium (Equations (1) and (4)). The only difference is that Figure 1a studies the solubility of the crystallizing component in liquid water as a function of temperature, while Figure 1b studies the influence of relative humidity on the state of the crystal. As shown in Section 2 (Equations (1) and (4)), the crystal solubility is directly linked to the deliquescence via the solid–liquid–vapor equilibrium.

If a mixture of crystalline fructose and liquid water at a composition left of the solubility line (Figure 1a) is stored at a fix temperature, e.g., 298.15 K, this results in an unsaturated solution (L). At fructose concentrations right of the solubility line, a saturated liquid phase evolves and coexists with crystalline fructose (fructose crystals + L region in Figure 1a). The saturated liquid phase always has a composition located on the solubility line (w_fructose_ = 0.78 at 298.15 K). The water activity of that saturated liquid phase is determined by the RH in the vapor phase according to Equation (4) in thermodynamic equilibrium. This RH is the DRH of fructose at the considered temperature and is shown in Figure 1b (61.5% RH at 298.15 K). 

Figure 1b shows the phase transitions occurring starting from crystalline fructose as a function of RH. When crystalline fructose is exposed to an RH below the DRH, crystalline fructose remains stable. If the RH in the atmosphere reaches DRH, water can be absorbed leading to a saturated fructose solution (having the composition on the solubility line in Figure 1a at that temperature) next to the fructose crystals. However, the equilibrium amount of absorbed water is not instantaneously reached, but the kinetics of this process depends on the driving force of the water sorption, whereas the latter depends on the ratio between the RH in the atmosphere and the DRH. If the RH is increased above the DRH, an unsaturated solution is formed (dissolution of all fructose crystals) with a water activity being identical to the RH of the equilibrium vapor phase. Only at DRH, crystalline fructose can coexist simultaneously with a vapor phase and a liquid phase in thermodynamic equilibrium. If the RH is decreased below the DRH, the liquid phase evaporates, and fructose crystallizes. The literature values for the DRH of fructose from 293.15 to 313.15 K agree with the PC-SAFT predicted DRH at room temperature. Above 303.15 K, the prediction slightly underestimates the experimental DRH values. This might result from deviations between the prediction and reality, but it might also be a result from kinetic inhibitions during the vapor-sorption measurement. The measured equilibrium water activity of a saturated solution at 298.15 K shows that this effect occurs at least at this temperature and therefore is likely to occur for higher temperatures as well.

**Figure 2:**
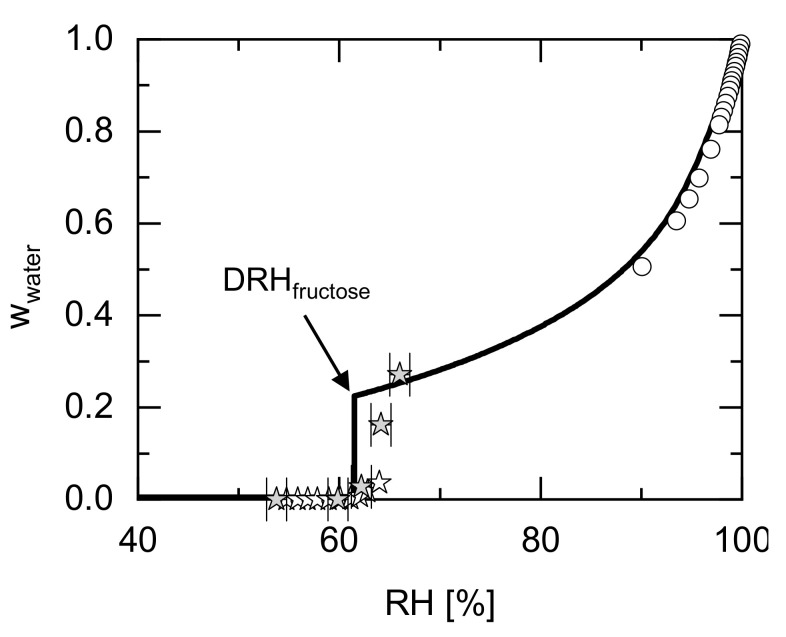
PC-SAFT-predicted water sorption of fructose as a function of RH at 298.15 K. Grey stars [45] and white stars [14] represent sorption data from literature. Equilibrium water activity measurements of fructose solutions obtained from the literature are shown as circles [46]. The thick line is the PC-SAFT-predicted water sorption in thermodynamic equilibrium. Experimental errors for the measurements are indicated or are smaller than symbol size.

Figure 2 shows the water sorption of crystalline fructose as a function of RH. In agreement with the observations from Figure 1 (pure fructose crystals below DRH, liquid solution above DRH), the predicted water sorption below DRH is negligible, whereas at a DRH of 61.5%, the water sorption is predicted to suddenly increase to w_water_ = 0.22. This value corresponds to the concentration of the saturated fructose/water solution ($w_{\mathrm{water}}=0.22=1-w_{\mathrm{fructose}}^{\mathrm{SL}}$). Above the DRH, the amount of absorbed water further increases, and the additional water from the humid atmosphere further dilutes the fructose/water solution. At 100% RH, fructose is predicted to be infinitely diluted by water from the vapor phase (w_water_→1). 

The sorption measurements [14,45] shown in Figure 2 indicate that the water sorption below 61% RH is almost zero and thus fructose crystals are stable below this RH. Above 61% RH, a water-sorption increase is observed. The experimental mass fraction of water slightly above DRH steadily increases from 0 to 0.23, while the water sorption is expected to jump abruptly to the water mass fraction of 0.22 according to thermodynamic phase equilibrium conditions. However, due to kinetic inhibitions, this abrupt jump to equilibrium water mass fractions is not observed in the measurements. Therefore, the prediction might indicate that the measured data points between 62 and 64% RH were not in thermodynamic equilibrium yet. Only the measured water sorption at 66% RH (highest investigated RH) seems to have reached thermodynamic equilibrium. 

Equilibrium water-activity measurements were performed for high water concentrations and high water activities [46]. These values are located at the top-right side of the diagram. In this part of the diagram, the water content increases steadily up to w_water_ = 1 at 100% RH. The equilibrium water-activity measurements of diluted fructose/water solutions are in almost quantitative agreement with the PC-SAFT predictions.

To further validate the prediction capability of PC-SAFT, DRH values of several single components from literature are compared to the predicted values listed in Table 4. The experimentally determined DRH values are separated in DRH and $a_{water}$ values according to their experimental source: DRH values were obtained from gravimetric vapor-sorption measurements, whereas $a_{water}$ values were obtained from measuring the relative humidity above a saturated solution (equilibrium water-activity measurement). DRH values determined via gravimetric sorption techniques are usually higher than the $a_{water}$ values, since the vapor sorption is kinetically inhibited at RHs slightly above DRH [2]. This is also observed for the values given in Table 4. The equilibrium water activity measurements thus allow for the determination of the DRH value without kinetic hindrance. However, they cannot measure the DRH of metastable crystals, as transformation to the thermodynamically stable form usually occurs. This can be better accomplished by gravimetric vapor sorption. 

In case of lactose, the measured $a_{water}$ is higher than the DRH determined via gravimetric vapor sorption due to hydrate formation at 298.15 K [44]. The hydrate has a lower aqueous solubility than the anhydrate leading to a higher water activity in the hydrate-saturated liquid phase compared to the anhydrate-saturated liquid phase.

CA forms a monohydrate or anhydrate depending on the RH and temperature. The solubility-phase diagram of CA is shown in the supporting information (Figure S1). CA anhydrate is not thermodynamically stable in water at 298.15 K, as the CA monohydrate is the stable form above 60.3% RH at 298.15 K [33]. Thus, CA anhydrate in contact with water transforms to CA monohydrate at 298.15 K, and therefore, the water activity in equilibrium becomes the water activity of a saturated solution of CA monohydrate (78% RH). The measured equilibrium values $a_{water}$ of CA anhydrate and CA monohydrate are equal (78% RH), because the CA anhydrate transforms to the CA monohydrate during measurement. Nevertheless, due to nonequilibrium conditions during the measurement, the DRH of CA anhydrate was measured using gravimetric vapor sorption.

Table 4 also compares the PC-SAFT predictions to those applying Raoult’s law. These two methods are based on solubility measurements of pure components in water. The difference between the predicted DRH values form Raoult’s law and PC-SAFT results from the water activity coefficient, which is only accounted for by PC-SAFT.

The average relative deviation ARD of N DRH values was calculated using the experimentally determined value ${\mathrm{DRH}}_{i}^{\exp}$ and the predicted value ${\mathrm{DRH}}_{i}^{\mathrm{pred}}$ in Equation (13).

(13) $$\mathrm{ARD}=\frac{100\%}{N}\cdot \overset{N}{\underset{i}{\sum }}\frac{\left|{\mathrm{DRH}}_{i}^{\exp}-{\mathrm{DRH}}_{i}^{\mathrm{pred}}\right|}{{\mathrm{DRH}}_{i}^{\exp}}$$

The ARD values of DRH/$a_{water}$ when applying Raoult’s is about 8%, whereas as the ARD of PC-SAFT predicted DRH values compared to the measured ones is only 2%. This indicates the importance of considering the activity coefficients for the prediction of the DRH values. The largest difference between the PC-SAFT predicted DRH value and the literature DRH value occurs for sucrose. For all other components, the deviation is below ±1.6% RH.

### 3.2. DRH Prediction of Multicomponent Crystal Mixtures

#### 3.2.1. DRH of a Binary Fructose/Glucose Crystal Mixture—Influence of the Crystal Ratio

**Figure 3:**
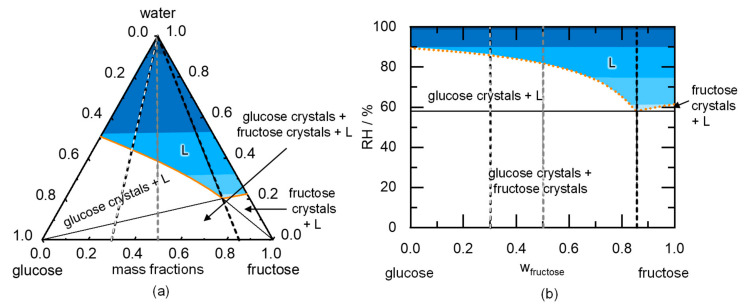
Predicted phase diagrams indicating the water deliquescence behavior of a glucose/fructose crystal mixture at 298.15 K. (**a**) Ternary-phase diagram indicating the PC-SAFT-predicted solubilities of glucose and fructose in water as thick solid lines and (**b**) PC-SAFT-predicted DRH as a function of fructose content in the fructose/glucose mixture shown as dotted lines. Darker shaded regions represent higher RH values (58.1–99% RH). The dashed lines represent the compositions investigated for water sorption in Figure 4.

Whereas the DRH prediction of single components is performed by solving two equilibrium conditions (Equations (1) and (4)), N + 1 equilibrium conditions must be solved for predicting DRH values of crystal mixtures with N crystals in physical contact (N times the Equations (1) and (4)). Figure 3 indicates the predicted phase diagram of a fructose/glucose crystal mixture. 

This example system is used to illustrate the phase diagrams and the link between deliquescence and water sorption. Pure water, fructose, and glucose are to be found in the corners of the triangle in Figure 3a. The solubility lines of glucose and fructose start on the left and right axis of the diagram, respectively, and intersect at the eutectic point. An aqueous glucose/fructose solution (L) is present above the solubility lines, whereas glucose crystals (glucose + L), fructose crystals (fructose + L), or both (glucose + fructose + L) coexist with saturated solutions below the solubility lines. The water activity in the liquid phase decreases from pure water (top of the triangle) from $a_{water}$ = 1 at $w_{water}$ = 1 downwards (decreasing color intensity in Figure 3a). The lowest water activity leading to a thermodynamically stable liquid phase is found at the eutectic point. This water activity corresponds to the DRH of the mixture (DRH_mix_) of glucose crystals and fructose crystals in contact with each other. The composition of the fructose/glucose crystal mixture at that point is the so-called eutonic composition, which is the same as the eutectic composition.

Figure 3b shows the phase behavior of a glucose/fructose crystal mixture upon exposure to RH. Analogous to the solubility line in Figure 3a, the deliquescence line in Figure 3b separates the region of a thermodynamically stable unsaturated solution above the line from the crystal phase below the solubility/deliquescence line. The glucose and fructose deliquescence lines start on the left and the right axis (predicted DRH_glucose_ and DRH_fructose_), respectively, and both decrease in the mixture. DRH_mix_ is obtained at the intersection of the deliquescence lines (with the eutonic or eutectic composition [14]). The water activity of the saturated liquid (composition of this liquid shown in Figure 3a) is fix and exactly corresponds to the DRH_mix_ shown in Figure 3b (see Equation (4)). 

Below the DRH_mix_ of glucose and fructose (58.1% RH predicted by PC-SAFT), glucose and fructose crystals are both thermodynamically stable, and there is never a liquid phase in thermodynamic equilibrium. At DRH_mix_ of the glucose/fructose mixture, besides glucose and fructose crystals, a liquid phase evolves exactly having the eutectic composition (see Figure 3a). For increasing RH, the composition of the initially eutectic liquid phase changes and is always to be found on the solubility line: For glucose/fructose compositions left of the eutectic composition, fructose dissolves with increasing RH (glucose + L); if the glucose/fructose crystal ratio is right of the eutectic composition, glucose dissolves (fructose + L). The composition of the crystal mixture does not have any influence on the DRH_mix_; only the RH above which every crystal is dissolved varies with the composition of the crystal mixture (according to the deliquescence line in Figure 3b). 

**Figure 4:**
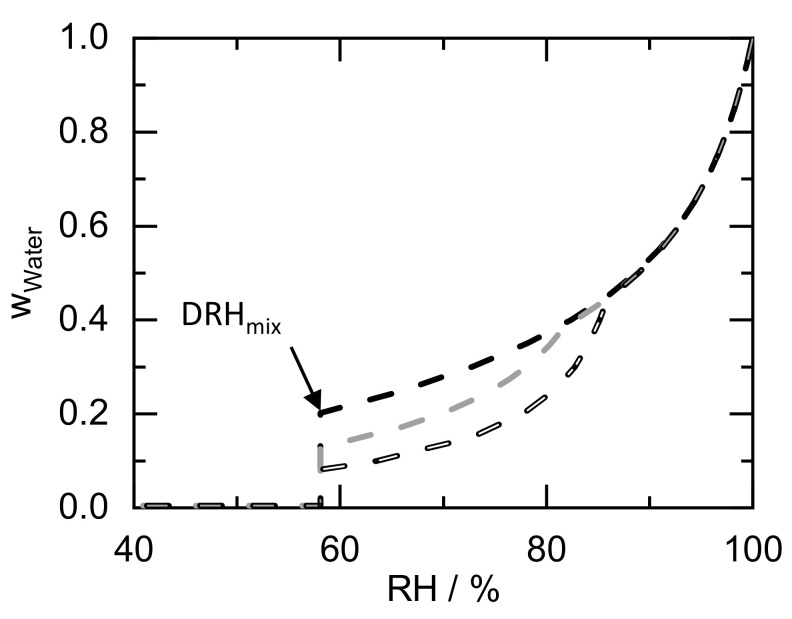
PC-SAFT-predicted water sorption of crystal mixtures with different fructose/glucose ratios as a function of RH at 298.15 K. The dashed lines are the PC-SAFT predicted water-sorption curves. Black dashed line is for w_fructose_ = 0.85; grey dashed lines is for w_fructose_ = 0.5; and white dashed line is for w_fructose_ = 0.3.

To further study the deliquescence of a crystal mixture, Figure 4 shows the predicted water sorption of a crystal mixture with different fructose/glucose ratios as a function of RH. In agreement to Figure 3b, the water sorption below DRH_mix_ (58.1% RH) is zero in all cases. Above DRH_mix_, the water sorption varies with varying composition of the crystal mixture, while water sorption always starts at the same RH (DRH_mix_ of 58.1% RH). 

The glucose/fructose crystal mixture with a mass fraction of w_fructose_ = 0.3 starts deliquescing at DRH_mix_. Above DRH_mix_, every crystal of fructose in contact either with a glucose crystal or in contact with the liquid phase dissolves in thermodynamic equilibrium (phase region of glucose crystals + L in Figure 3a). The water uptake can be calculated using the phase diagram shown in Figure 3a. The mass fraction of water is obtained from the intersection of the line connecting pure water with the crystal mixture ratio (white-dashed line in Figure 3) and the phase boundary between (glucose + L) and (glucose + fructose + L) (line connecting pure glucose and the eutectic composition). Thus, this crystal mixture takes up w_water_ = 0.08 water in thermodynamic equilibrium, dissolving all fructose crystals and forming a saturated glucose mixture. The liquid phase glucose/fructose ratio increases with increasing RH, starting from the eutectic point and following the glucose deliquescence line in Figure 3b until reaching a water sorption of w_water_ = 0.44 and 85.9% RH (at w_fructose_ = 0.3). An unsaturated solution occurs above this RH. Higher RH leads to more water absorbed into the liquid phase and further dilution of the solute components. At 100% RH, fructose and glucose are predicted to be infinitely diluted by water from the vapor phase (w_water_ = 1). 

The w_fructose_ = 0.5 crystal mixture shows a higher water sorption at the DRH_mix_ of w_water_ = 0.13 compared to the w_fructose_ = 0.3 crystal mixture. Furthermore, the last crystal dissolves at a lower RH of 81.8% RH with w_water_ = 0.39 (compare Figure 3a). At the eutectic composition (w_fructose_ = 0.85), fructose and glucose equally deliquesce at the DRH_mix_ leading to a water sorption of w_water_ = 0.2. An unsaturated solution evolves above this RH. The RH above which every crystal of the crystal mixture dissolves is represented by the deliquescence line in Figure 3b. The fact that the water sorption for the different compositions of glucose/fructose is almost equal above a certain RH (85.9% RH for the considered compositions) is due to the horizontal course of the iso-RH-lines in Figure 3a. For systems with different behavior, the water sorption of the unsaturated solution might vary for different compositions of the crystal mixture.

#### 3.2.2. DRH Values of Binary, Ternary and Quaternary Crystal Mixtures

Table 5 shows predicted DRH_mix_ values for binary, ternary, and quaternary mixtures of crystals. 

The experimentally determined DRH_mix_ values were obtained from literature. Some equilibrium $a_{water}$ measurements are marked with a star in order to indicate that this crystal is not thermodynamically stable according to our prediction, e.g., depending on the measured DRH value, either the CA anhydrate (below 60.3% RH at 298.15 K) or the CA hydrate (above 60.3% RH at 298.15 K) is thermodynamically stable. In the event that deliquescence occurs below 60.3% RH, the CA anhydrate mixture DRH can be measured in thermodynamic equilibrium; while above 60.3% RH, the CA hydrate mixture DRH is measured in thermodynamic equilibrium.

For most crystal mixtures, the calculated DRH values using Raoult’s law are higher compared to the PC-SAFT predicted values. This means, that there are RH regions at which Raoult’s law predicts the crystal mixture as being stable, while deliquescence already occurs. The ARD of the predicted DRH values of all investigated crystal mixtures using Raoult’s law differ by 15% from the experimental DRH values (determined via gravimetric vapor sorption) [2,14,44,45]. The ARD of PC-SAFT predicted DRH values compared to the measured ones [2,14,44,45] is only 9%. This again shows the importance of considering the nonideal interactions between the components, which are considered by the activity coefficients. It is worth mentioning, that the interactions between the two solutes in the liquid phase are fully predicted using PC-SAFT. Thus, the ARD of 9% for the PC-SAFT predicted DRH values reveals a satisfying agreement to the literature DRH values. In Table 5, the largest deviations between the PC-SAFT-predicted DRH and literature DRH were obtained for crystal mixtures with sucrose. This results from the discrepancy in the sucrose aqueous solubility (compare Section 3.1). Excluding the sucrose mixtures, the predicted DRH values agree within ±7.6% RH. Seeing as the scope of this work is not the exact correlation of experimentally determined DRH values but predicting the DRH of crystal mixtures based on a minimum amount of experimental data, these are quite good results.

### 3.3. Predicting DRH as a Function of Temperature

**Figure 5:**
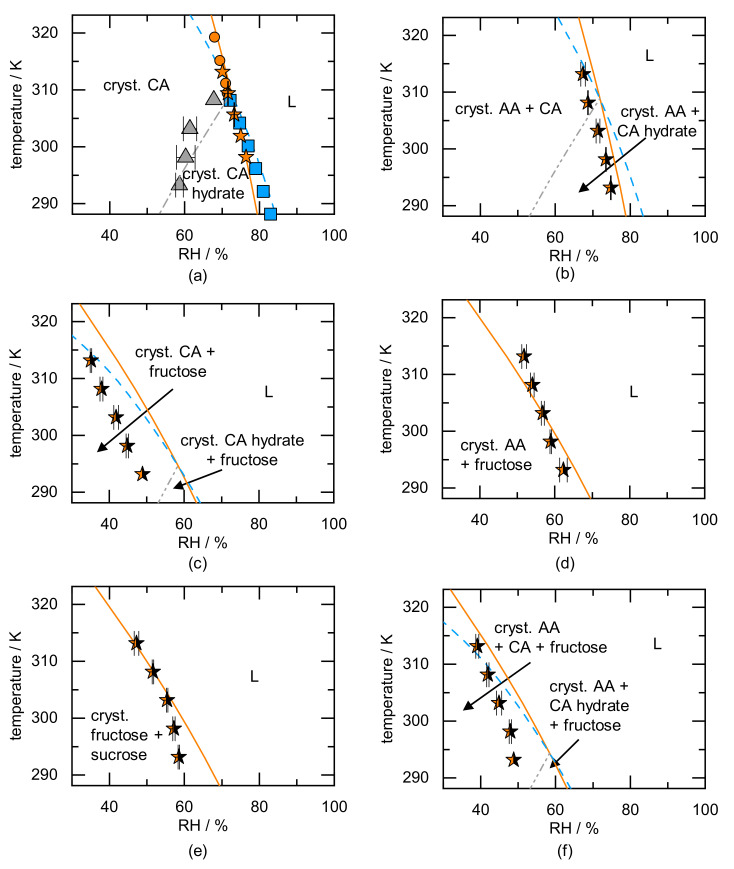
Predicted temperature-dependent DRH of (**a**) CA and for mixtures of (**b**) AA/CA, (**c**) CA/fructose, (**d**) AA/fructose, (**e**) fructose/sucrose, and (**f**) AA/CA/fructose. The equilibrium water-activity data (**a**) of CA hydrate are shown as squares; CA anhydrate are shown as circles [33]. Gravimetric vapor-sorption measurements of CA anhydrate in (**a**) are shown as stars [44]. The triangles in (**a**) indicate data from Reference [33] showing the solid–solid transformation of CA anhydrate to hydrate. Gravimetric vapor-sorption measurements of crystal mixtures in (**b**–**f**) are shown as half-filled stars [44]. The solid lines in (**a**–**f**) show the predicted DRHs of the anhydrate, whereas the dashed lines show the predicted DRHs of the hydrate (where applicable). The dash-dotted lines mark the predicted solid–solid transformation from anhydrate to hydrate.

The DRH of (a) pure CA, (b) AA/CA, (c) CA/fructose, (d) AA/fructose, (e) fructose/sucrose, and (f) AA/CA/fructose as a function of temperature is shown in Figure 5. 

The phase behavior of CA (anhydrate and hydrate) is shown in Figure 5a. The equilibrium water activity measurements obtained from Reference [33] show that CA hydrate is stable below a temperature of 309 K (squares in Figure 5a). CA anhydrate is the stable form above this temperature (circles in Figure 5a). The gravimetric vapor-sorption measurements of CA anhydrate obtained from Reference [44] (stars in Figure 5a) agree with the equilibrium water activity measurements above the transition temperature. Below the transition temperature, the gravimetric vapor-sorption measurements differ from the equilibrium water activity measurements. This is an indirect proof that hydrate formation indeed occurs in thermodynamic equilibrium, but the anhydrate did not fully transform to the hydrate during the gravimetric vapor-sorption measurement. We conclude that the DRH of the metastable form (CA anhydrate) was observed without knowing that this is the metastable form [44]. The DRH values obtained from gravimetric vapor-sorption measurements (stars in Figure 5a) agree with the predicted CA anhydrate deliquescence line above and below the transition temperature.

The predicted deliquescence lines of CA anhydrate and hydrate intersect at the transition temperature (309.45 K). Below the transition temperature, the predicted DRH of CA hydrate is higher than that of the CA anhydrate. Above the transition temperature, the predicted DRH of CA hydrate is lower than that of the CA anhydrate. At RHs above the DRH of the crystal mixture, a liquid forms (region L). It can be seen that the DRH values obtained from equilibrium water activity measurements (circles and squares in Figure 5a) are in excellent agreement with the predicted DRH values for the respective form. 

The CA anhydrate–hydrate solid–solid transformation RH was obtained for different temperatures from Reference [33]. The measurements indicate that the anhydrate is stable at low RHs and at higher temperatures. The predicted solid–solid transformation line (grey dash-dotted line) separates the region of stable CA anhydrate from the CA hydrate formation region and is in agreement with the experimentally determined solid–solid transition between hydrate and anhydrate. The ARD of the predicted solid–solid transformation RH from the experimental one is 4%. According to the prediction, the hydrate is not thermodynamically stable above 309.45 K. 

The RH influence on a CA/AA crystal mixture is studied in Figure 5b. The gravimetric vapor-sorption measurement was performed with a CA anhydrate/AA crystal mixture at different temperatures [44]. Although CA hydrate is the stable form below 309.45 K (compare grey dash-dotted line in Figure 5b), we assume that the transformation of CA anhydrate to the CA hydrate did not occur during the performed measurements (hydrate formation of pure CA did not occur during measurement either, see Figure 5a). The experimental data in Figure 5b matches with the predicted deliquescence line of the CA anhydrate/AA crystal mixture. Comparing the predicted CA hydrate deliquescence line in Figure 5a,b, it can be seen that the AA influence on the DRH of the CA hydrate is negligible.

The temperature-dependent deliquescence of the CA/fructose mixture is shown in Figure 5c. The experimental data [44] indicate that deliquescence occurs for RHs lower than 48.9% for temperatures above 293.15 K. This results from the low DRH value of fructose (61% RH at 298.15 K [14]). In this case, the presence of fructose prevents the formation of CA hydrate above 293.15 K, since deliquescence occurs prior to hydrate formation (hydrate formation above 59% RH at 293.15 K [33]). The predicted deliquescence line of CA anhydrate/fructose overestimates the measured DRH. Nevertheless, the course of the measured DRH values for the CA/fructose agree with the predicted deliquescence line of CA/fructose. The predicted CA hydrate deliquescence line in Figure 5c is at significantly lower RH compared to the system without fructose (Figure 5a). The CA hydrate in presence of fructose deliquesces prior to the CA hydrate in presence of AA (compare Figure 5b). This results from the low DRH of fructose compared to AA and the resulting lower DRH_mix_ of the crystal mixture. The temperature of the intersection of CA anhydrate/fructose and CA hydrate/fructose deliquescence line explicitly does not mean that the CA hydrate cannot be present above this temperature. The CA hydrate might still be thermodynamically stable above the solid–solid transformation RH of hydrate formation as soon as fructose is dissolved entirely (compare presence of glucose at RH above DRH_mix_ in Figure 3b).

The system AA/fructose (Figure 5d) looks simpler, as hydrate formation does not occur. Thus, below the DRH, crystals of both AA and fructose are stable and above the deliquescence line a solution forms. The measured DRH value matches the predicted one at 303.15 K. Above this temperature, the predicted DRH is slightly higher than the measured one, and below this temperature, the predicted DRH is slightly lower than the measured one with an overall ARD of 4%. The system fructose/sucrose (Figure 5e) does not form any hydrates either for the here-investigated conditions. The predicted deliquescence line agrees with the measured DRH values at temperatures above 303.15 K. Below that temperature, the prediction differs by 9% from the measured DRH values.

The temperature dependence of deliquescence for the AA/CA/fructose ternary crystal mixture is finally shown in Figure 5f. The literature data show that the crystal mixture deliquesce above 48.8% at 293.15 K [44]. This leads to the fact that, similar to the CA/fructose crystal mixture, hydrate does not occur (hydrate formation of CA hydrate above 59% RH [33]). The predicted DRH of the ternary mixture is higher than the one obtained from Reference [44] (overall ARD 11%). Particularly considering that all lines in Figure 5 were full predictions using solubility data of each individual component in water only, the predictions seem valuable for estimating stability regions. Thus, the RH and temperature limits for stable food or pharmaceutical ingredients (also in crystal mixtures) can be predicted, and time-consuming experiments (vapor-sorption measurements or equilibrium water activity measurements) can be prevented. The predicted phase diagrams can be used to determine which component is responsible for the low DRH_mix_ in case of crystal mixtures, and the outcome of the deliquescence (complete dissolution of either of the components) can be predicted as a function of the crystal mixture composition.

The temperature-dependent deliquescence behavior of anhydrates as well as of hydrates and even of crystal mixtures thereof can be quantitatively predicted via PC-SAFT. The prediction explicitly differentiates between the solid state (anhydrate vs. hydrate, see Equations (1) and (2)). The DRH can be easily predicted as a function of temperature as well as for multicomponent crystal mixtures (the decrease in DRH of crystal mixtures does not depend on its composition). 

Moreover, combining components with a low DRH and hydrate-forming components can prevent hydrate formation, because the crystals deliquesce before hydrate formation can occur.

## 4. Conclusions

Thermodynamic-phase diagrams were used to predict deliquescence relative humidity (DRH) values of crystals. Moreover, the DRH decrease in physical crystal mixtures and the influence of temperature was successfully investigated. The DRH was predicted by a coupled solubility/water-sorption calculation with PC-SAFT, which explicitly considers intermolecular interactions between the components in the liquid phase. By comparing the DRH values predicted via PC-SAFT with those calculated by Raoult’s law, it was shown that thermodynamic activity coefficients are indispensable for a correct prediction of the deliquescence behavior. 

Predicted DRH values of single crystals and of binary, ternary, and quaternary crystal mixtures were found in good agreement with experimental data. The overall average relative deviation of measured and PC-SAFT-predicted DRH values was 2% for single crystals and 9% for multicomponent crystal mixtures. Furthermore, the prediction can distinguish between DRH values of hydrates and anhydrates as well as predict the DRH of metastable crystals. In particular, the addition of crystals with low DRH to hydrate-forming components can prevent the hydrate formation as deliquescence occurs before hydrate formation is possible. For example, hydrate formation of citric acid (above 59% RH at 293.15 K) could be prevented by the addition of fructose (DRH_mix_ of 48.9% RH at 293.15 K).

Based on easily accessible solubility data of single crystals in water, a broad variety of humidity risk attributes for crystals was predicted via PC-SAFT, such as DRH values, the temperature influence on deliquescence, hydrate formation, water sorption, and the mutual influence of crystals on the deliquescence behavior of their mixtures. Thus, the stability of crystals and crystal mixtures against RH can be predicted without the need of additional time-consuming experiments.

## Figures and Tables

**Table 1:** PC-SAFT pure-component parameters of the components investigated in this work.

Component	$M_{i}/g\;{\mathrm{mol}}^{-1}$	$m_{seg}\;M_{i}^{-1}/\mathrm{mol}\;g^{-1}$	$\sigma_{i}/\text{Å}$	$u_{i}\;k_{B}^{-1}/K$	$\epsilon^{A_{i}B_{i}}\;k_{B}^{-1}/K$	$\kappa^{A_{i}B_{i}}$	$N_{i}^{assoc}$	Ref.
water	18.02	0.06687	^A^	353.94	2425.67	0.0451 ^B^	1/1	[26]
ascorbic acid	176.13	0.06647	2.367	353.44	2600.68	0.039	4/1	[27]
citric acid	192.12	0.0445	2.723	227.18	2488	0.044	4/4	[28]
fructose	180.16	0.0410	2.849	237.19	5000	0.1	5/5	[29]
glucose	118.16	0.0368	2.986	244.53	5000	0.1	5/5	[29]
nicotinamide	122.12	0.0381	2.178	176.69	2195.3	0.02	2/2	[30]
lactose	342.3	0.0419	2.811	319.21	5000	0.1	8/8	[29]
saccharin	183.18	0.02393	4.125	383.54	853.03	0.02	1/1	[31]
sucrose	342.30	0.0435	2.827	297.39	5000	0.1	8/8	[29]

^A^ σ = 2.7927 + 10.11·exp(−0.01775·*T*) – 1.417·exp(−0.01146·*T*). ^B^ Typo in reference [26].

**Table 2:** PC-SAFT binary interaction parameters used in this work and the type of experimental data used for fitting.

Components	k_ij,T_/K^−1^	k_ij,b_	Fitted to	Reference for Parameters
ascorbic acid/water	-	−0.018	osmotic coefficients/density	[27]
citric acid/water	-	−0.07	solubility	this work ^B^
fructose/water	1.40 × 10^−4^	−0.097241 ^A^	osmotic coefficients/density	[29]
glucose/water	2.24 × 10^−4^	−0.119186 ^A^	osmotic coefficients/density	[29]
nicotinamide/water	9.46 × 10^−5^	−0.0294	solubility	[30]
lactose/water	1.84 × 10^−4^	−0.08696 ^A^	osmotic coefficients/density	[29]
saccharin/water	-	−0.01507	solubility	[31]
sucrose/water	2.56 × 10^−4^	−0.113426 ^A^	osmotic coefficients/density	[29]

^A^ k_ij,b_ values are stated at 298.15 K in the reference. ^B^ the modeling of the solubility-phase diagram is shown in the supporting information.

**Table 3:** Melting properties of the components investigated in this work.

Component	$T^{SL}/K$	$\Delta h^{SL}/\mathrm{kJ}\;{\mathrm{mol}}^{-1}$	$\Delta c_{p}^{SL}/J\;{\mathrm{mol}}^{-1}K^{-1}$	Ref.
ascorbic acid	465.15	29.12	43 [27]	[34]
citric acid	428.55	41.84	159.46 ^a^ [35]	[36]
fructose	353.2	32.4	-	[37]
glucose	408.2	32.5	-	[37]
nicotinamide	401.15	28.0	78.12 [38]	[39]
lactose	433.2	75.3	-	[37]
saccharin	502.9	32.1	-	[40]
sucrose	433.2	41.1	-	[37]

^a^ It was assumed that the heat capacity of the glassy state is similar to the heat capacity of the solid state [41].

**Table 4:** Comparison of predicted and measured DRH values of single components at 298.15 K. DRH values were predicted using PC-SAFT or using Raoult’s law. Measurements from the literature either show the equilibrium water activity of a saturated solution ($a_{water}$) or the DRH measured via gravimetric vapor sorption. In thermodynamic equilibrium, these values should be identical. The standard deviations from the references are added if reported.

	DRH Prediction/%		DRH Measurement/%		Reference of Measurement
Component(s)	Raoult	PC-SAFT	DRH	$a_{water}$	
Single components					
ascorbic acid (AA)	96.9	97.4	97.5 ± 1	97 ± 0	[44]
citric acid (CA)	86.0	76.4	75.0 ± 1	78 *	[14]
citric acid hydrate	87.4	79.4	78.0 ± 1	78	[14]
fructose	74.3	61.5	62.0 ± 1	61	[14]
glucose	91.1	89.4	91.0 ± 1	90	[14]
nicotinamide (NA)	89.6	93.6	94.5 ± 0.3		[45]
lactose	95.0	94.2	95.0 ± 1	97	[14]
saccharin	100.0	100.0			-
sucrose	94.1	92.0	85.0 ± 1	85	[14]

* value considered as error prone due to nonequilibrium conditions as anhydrate CA is unstable at these conditions.

**Table 5:** Comparison of predicted and measured DRH values for multicomponent crystal mixtures at 298.15 K. DRH values were predicted using PC-SAFT or using Raoult’s law. Measurements from literature either show the equilibrium water activity ($a_{water}$) or the DRH measured via dynamic vapor sorption. From a thermodynamic point of view, these values should be equal. The standard deviations from the references are added if reported.

	DRH Prediction/%		DRH Measurement/%		Reference of Measurement
Component (s)	Raoult	PC-SAFT	DRH	$a_{water}$	
binary mixtures					
AA-CA	82.8	75.7	74.4 ± 1	73.5 ± 0.1 *	[44]
AA-CA hydrate	84.3	78.4	74	75	[2]
AA-fructose	71.2	61.3	58.9 ± 1	58.9 ± 0.2	[44]
AA-sucrose	91.0	87.7	85	83	[2]
CA-fructose	60.3	55.6	48.0 ± 1	47	[14]
CA hydrate-fructose	61.7	55.0	52	48 *	[2]
CA-glucose	77.1	72.4	68.0 ± 1	67 *	[14]
CA hydrate-glucose	78.5	74.3	-	68	[2]
CA-sucrose	80.1	75.3	64.0 ± 1	60 ^a^	[14]
CA hydrate-sucrose	81.6	77.8	65	56 *	[2]
fructose-glucose	65.4	58.1	60 ± 1	54	[14]
fructose-NA	63.9	49.1	55.3 ± 0.3	-	[45]
fructose-saccharin	74.3	61.5	61.5 ± 0.3	-	[45]
fructose-sucrose	68.5	61.1	58 ± 1	56	[14]
glucose-sucrose	85.3	86.5	79 ± 1	78	[14]
sucrose-NA	83.7	76.1	80 ± 0.3	-	[45]
ternary mixtures					
AA-CA-fructose	57.2	55.4	47.9 ± 1	47.9 ± 0.3	[44]
AA-CA hydrate-sucrose	78.4	76.5	56	56 *	[2]
CA-fructose-glucose	51.4	52.8	50 ± 1	43 ^a^	[14]
CA hydrate-fructose-glucose	52.8	51.9	-	47 *	[2]
CA-fructose-sucrose	54.4	55.3	55 ± 1	44 ^a^	[14]
CA-glucose-sucrose	71.2	71.6	64 ± 1	59 ^a^	[14]
fructose-glucose-sucrose	59.6	57.7	58 ± 1	53 ^a^	[14]
quaternary mixtures					
CA-fructose-glucose-sucrose	45.5	52.5	54 ± 1	48 ^a^	[14]
CA hydrate-fructose-glucose-sucrose	47.0	1.9	-	44 *	[2]

^a^ Equilibrium was not reached within the investigated 24 h according to the reference. * Measurement expected to be error prone since crystal form is metastable according to PC-SAFT prediction.

## Data Availability

Data is contained within the article and Supplementary Material.

## Supplementary Materials

Modelling of highly soluble hydrates, Figure S1: Temperature-dependent binary-phase diagrams of fructose and water.

## Sample Availability

Samples of the compounds are not available from the authors.

## Notation

a	thermodynamic activity
A	Helmholtz energy/J
$\Delta c_{P,i}^{SL}$	difference of the heat capacity of the solid and the liquid component *i* at its melting point/kJ K^−1^ kg^−1^
${\Delta h}^{\mathrm{SL}}$	melting enthalpy/kJ kg^−1^
$k_{b}$	Boltzmann constant/J K^−1^
$k_{ij}$	binary interaction parameter
$m_{seg}$	number of segments of component *i*
$M_{i}$	molar mass of component *i*/g mol^−1^
N	number experimental data points
$N_{i}^{assoc}$	number of association sites
p	pressure/bar
R	gas constant/J mol^−1^ K^−1^
T	temperature/K
$u_{i}/k_{B}$	dispersion energy parameter/K
$w_{i}$	mass fraction of component *i*
$x_{i}$	mole fraction of component *i*
Greek symbols	
$\;\gamma_{i}$	activity coefficient of component *i*
$\varepsilon^{AiBi}/k_{B}$	association energy parameter/K
$\kappa^{AiBi}$	association volume parameter
ρ	density/g cm^−3^
$\nu_{i}$	stoichiometric coefficient of component *i*
$\sigma_{i}$	segment diameter/Å
Subscripts	
calc	calculated
exp	experimental
i, j	component *i*, component *j*
Superscripts	
L	liquid
trans	transition
S	solid
V	vapor
Abbreviations	
AA	ascorbic acid
ARD	average relative deviation
CA	citric acid anhydrate
DRH	deliquescence relative humidity
L	liquid phase
PC-SAFT	perturbed-chain statistical associating fluid theory
RH	relative humidity
