# Supplementary material for: Predicting Deliquescence Relative Humidities of Crystals and Crystal Mixtures
Source: Molecules. 2021 May 26;26(11):3176. doi: 10.3390/molecules26113176 (PMC8198525; doi:10.3390/molecules26113176)
Supplement: Supplementary file 1 [file molecules-26-03176-s001.zip › molecules-1222801-supplementary.pdf]

Predicting deliquescence relative humidities of crystals and crystal mixtures

*Heiner Veith, Christian Luebbert, Gabriele Sadowski**

TU Dortmund University, Department of Chemical and Biochemical Engineering, Laboratory of Thermodynamics, Emil-Figge-Str. 70, D-44227 Dortmund, Germany

1. Modeling of highly soluble hydrates

To model hydrate solubilities (solvates can be described in the same way), three equilibrium reactions have to be considered.

I. The first equilibrium reaction is the solid-liquid equilibrium between an anhydrate component (comp) and a solvent. As the solvent is not involved in this reaction, it can be left out in the equilibrium reaction I.

$$comp^S \xleftrightarrow{K_{comp}} comp^L \tag{I}$$

The solid phase is pure, thus the activity in the solid phase is unity. This leads to the activity-based equilibrium constant K_{comp} as defined in Eq. (S1) (Prausnitz et al., 1999).

$$K_{comp} = a_i = x_i \cdot \gamma_i = \exp\left[-\frac{\Delta h_i^{SL}}{R \cdot T} \cdot \left(1 - \frac{T}{T_i^{SL}}\right) - \frac{\Delta c_{p,i}^{SL}}{R} \cdot \left(ln\left(\frac{T_i^{SL}}{T}\right) - \frac{T_i^{SL}}{T} + 1\right)\right] \tag{S1}$$

In this equation, T_i^{SL} is the melting temperature, Δh_i^{SL} is the melting enthalpy, and $\Delta c_{p,i}^{SL}$ is the change of heat capacity of the component i upon melting. R is the ideal gas constant and T is the temperature. a_i is the activity of the component i in the liquid phase.

II. The second equilibrium reaction is the hydrate-formation reaction from its anhydrate (comp) with the stoichiometry ν_i and ν_{water}.

$$hydrate^S \xleftrightarrow{K_{h,hydrate}} \nu_i\ comp^S + \nu_{water}\ water^L \tag{II}$$

The solid components are assumed to crystallize as pure components. Thus, the activity of each solid (comp and hydrate) is unity. Therefore, the equilibrium constant $K_{h,hydra}$ is determined by the activity of water a_{water} in the liquid phase. Below this water activity, which is temperature dependent, the anhydrate is stable and above this water activity, the hydrate is the thermodynamically stable form. The water activity can be calculated by the extended Clausius-Clapeyron equation, which was developed by the group of *Grant (Brittain et al., 2009; Gu and Grant, 2001)*. In this work, it was extended by the water activity at transition temperature a^{trans} to be able to describe highly-soluble hydrates.

$$K_{h,hydrate} = a_{water}^{\nu_{water}} = \left(a^{trans} \cdot exp\left[\frac{-\Delta h^{trans}}{\nu_{water} \cdot R \cdot T}\left(1 - \frac{T}{T^{trans}}\right)\right]\right)^{\nu_{water}} \tag{S2}$$

In this equation, a^{trans} is the water activity and Δh^{trans} is the hydrate transition enthalpy at the transition temperature T^{trans}.

III. The equilibrium reaction finally considered is the dissolution of hydrate in water (dissociation of the hydrate into the component and water).

$$hydrate^{S} \xleftrightarrow{K_{s,hydrate}} \nu_i\ comp^{L} + \nu_{water}\ water^{L} \tag{III}$$

Assuming the hydrate thermodynamic activity in the solid phase being unity leads to the activity-based equilibrium constant Eq. (S3).

$$K_{s,hydrate} = a_i^{\nu_i} \cdot a_{water}^{\nu_{water}} \tag{S3}$$

Reactions I and II are combined to obtain the equilibrium reaction III. Thus, the equilibrium constant of reaction III can be calculated using the component activity and water activity of Eq.(S1) and Eq.(S2) to obtain Eq. (S4) (see also Eq. (3) in the manuscript).

$$K_{s,hydrate} = \left(exp\left[-\frac{\Delta h_i^{SL}}{R \cdot T}\left(1 - \frac{T}{T_i^{SL}}\right) - \frac{\Delta c_{P,i}^{SL}}{R}\left(ln\left(\frac{T_i^{SL}}{T}\right) - \frac{T_i^{SL}}{T} + 1\right)\right]\right)^{\nu_i} \cdot \left(a^{trans} \cdot exp\left[\frac{-\Delta h^{trans}}{\nu_{water} \cdot R \cdot T}\left(1 - \frac{T}{T^{trans}}\right)\right]\right)^{\nu_{water}} \tag{S4}$$

For hydrates with an extremely low water solubility, the a^{trans} is close to unity and can therefore be neglected.

2. Modeling the citric acid/water phase diagram

The modeling of the system citric acid (CA)/water was performed by fitting the calculated solubility of anhydrate and hydrate to the measured solubility of anhydrate and hydrate (Groen and Roberts, 2001), by varying the k_{ij} between CA and water. The water activity at transition temperature of 309.45 K a^{trans} was iteratively determined to 0.725 during the modeling. The transition enthalpy of 10.85 kJ mol^{-1} (Apelblat, 1986) and a transition temperature of 309.45 K (Allan and Mauer, 2017) was used for that.

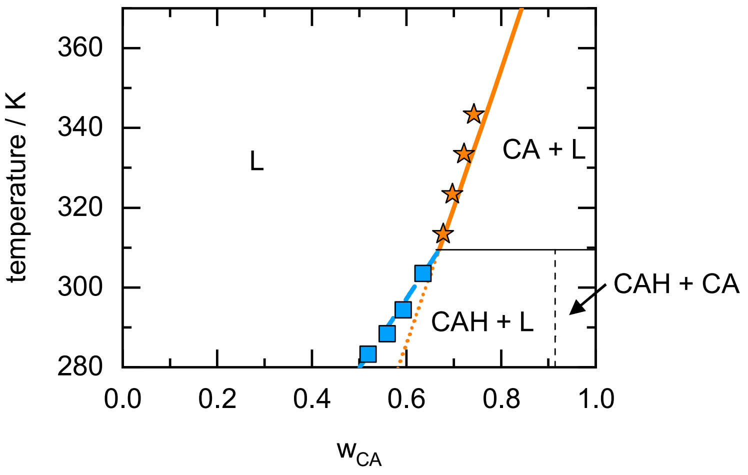

Figure S1: Temperature dependent binary phase diagrams of fructose and water. The solid line is the modeled equilibrium solubility of CA anhydrate in water, the dashed line is the hydrate solubility line in water. The stars and squares are CA anhydrate and CA hydrate (CAH) solubility data (Groen and Roberts, 2001).

References

Allan, M., Mauer, L.J., 2017. RH-temperature phase diagrams of hydrate forming deliquescent crystalline ingredients. Food Chem. 236, 21–31.

Apelblat, A., 1986. Enthalpy of solution of oxalic, succinic, adipic, maleic, malic, tartaric, and citric acids, oxalic acid dihydrate, and citric acid monohydrate in water at 298.15 K. J. Chem. Thermodyn. 18 (4), 351–357.

Brittain, H.G., Grant, D.J.W., Myrdal, P.B., 2009. Effects of Polymorphism and Solid-State Solvation on Solubility and Dissolution Rate, in: Brittain, H.G. (Ed.), Polymorphism in pharmaceutical solids, 2nd ed. ed. Informa Healthcare, New York, United States.

Groen, H., Roberts, K.J., 2001. Nucleation, Growth, and Pseudo-Polymorphic Behavior of Citric Acid As Monitored in Situ by Attenuated Total Reflection Fourier Transform Infrared Spectroscopy. J. Phys. Chem. B 105 (43), 10723–10730.

Gu, C.-H., Grant, D.J., 2001. Estimating the relative stability of polymorphs and hydrates from heats of solution and solubility data. J. Pharm. Sci. 90 (9), 1277–1287.

Prausnitz, J.M., Lichtenthaler, R.N., de Azevedo, E.G., 1999. Molecular thermodynamics of fluid-phase equilibria, 3rd ed. ed. Prentice Hall international series in the physical and chemical engineering, Upper Saddle River, NJ.
